# Development of Inflammatory Bowel Disease Is Linked to a Longitudinal Restructuring of the Gut Metagenome in Mice

**DOI:** 10.1128/mSystems.00036-17

**Published:** 2017-09-05

**Authors:** Thomas Sharpton, Svetlana Lyalina, Julie Luong, Joey Pham, Emily M. Deal, Courtney Armour, Christopher Gaulke, Shomyseh Sanjabi, Katherine S. Pollard

**Affiliations:** a Department of Microbiology, Oregon State University, Corvallis, Oregon; b Department of Statistics, Oregon State University, Corvallis, Oregon; c Gladstone Institutes, San Francisco, California, USA; d Department of Microbiology & Immunology, University of California, San Francisco, San Francisco, California, USA; e Department of Epidemiology & Biostatistics, Institute for Human Genetics, and Institute for Computational Health Sciences, University of California, San Francisco, San Francisco, California, USA; University of Chicago

**Keywords:** inflammatory bowel disease, lipooligosaccharide transporter, longitudinal, metagenomics, protein function, statistics

## Abstract

The gut microbiome is linked to inflammatory bowel disease (IBD) severity and altered in late-stage disease. However, it is unclear how gut microbial communities change over the course of IBD development, especially in regard to function. To investigate microbiome-mediated disease mechanisms and discover early biomarkers of IBD, we conducted a longitudinal metagenomic investigation in an established mouse model of IBD, where damped transforming growth factor β (TGF-β) signaling in T cells leads to peripheral immune activation, weight loss, and severe colitis. IBD development is associated with abnormal gut microbiome temporal dynamics, including damped acquisition of functional diversity and significant differences in abundance trajectories for KEGG modules such as glycosaminoglycan degradation, cellular chemotaxis, and type III and IV secretion systems. Most differences between sick and control mice emerge when mice begin to lose weight and heightened T cell activation is detected in peripheral blood. However, levels of lipooligosaccharide transporter abundance diverge prior to immune activation, indicating that it could be a predisease indicator or microbiome-mediated disease mechanism. Taxonomic structure of the gut microbiome also significantly changes in association with IBD development, and the abundances of particular taxa, including several species of *Bacteroides*, correlate with immune activation. These discoveries were enabled by our use of generalized linear mixed-effects models to test for differences in longitudinal profiles between healthy and diseased mice while accounting for the distributions of taxon and gene counts in metagenomic data. These findings demonstrate that longitudinal metagenomics is useful for discovering the potential mechanisms through which the gut microbiome becomes altered in IBD.

**IMPORTANCE** IBD patients harbor distinct microbial communities with functional capabilities different from those seen with healthy people. But is this cause or effect? Answering this question requires data on changes in gut microbial communities leading to disease onset. By performing weekly metagenomic sequencing and mixed-effects modeling on an established mouse model of IBD, we identified several functional pathways encoded by the gut microbiome that covary with host immune status. These pathways are novel early biomarkers that may either enable microbes to live inside an inflamed gut or contribute to immune activation in IBD mice. Future work will validate the potential roles of these microbial pathways in host-microbe interactions and human disease. This study was novel in its longitudinal design and focus on microbial pathways, which provided new mechanistic insights into the role of gut microbes in IBD development.

## Abstract (precis)

IBD patients harbor distinct microbial communities with functional capabilities different from those seen with healthy people. But is this cause or effect? Answering this question requires data on changes in gut microbial communities leading to disease onset. By performing weekly metagenomic sequencing and mixed-effects modeling on an established mouse model of IBD, we identified several functional pathways encoded by the gut microbiome that covary with host immune status. These pathways are novel early biomarkers that may either enable microbes to live inside an inflamed gut or contribute to immune activation in IBD mice. Future work will validate the potential roles of these microbial pathways in host-microbe interactions and human disease. This study was novel in its longitudinal design and focus on microbial pathways, which provided new mechanistic insights into the role of gut microbes in IBD development.

## INTRODUCTION

Inflammatory bowel disease (IBD) is an increasingly prevalent chronic autoimmune disease in which the cells of the immune system attack intestinal tissue (1–3). Quality of life deteriorates, and patients die in severe cases. Unfortunately, the etiology of disease remains unclear and is likely complex (4). Discovery of the factors that contribute to IBD onset, development, and severity is needed to ensure accurate and effective health care. Epidemiological studies and animal model experiments have identified genetic factors (5–7) and lifestyle factors that associate with IBD, including diet (8) and exercise (9). But these factors are not precise predictors of disease risk, severity, or response to treatment, and many questions remain regarding disease mechanisms. Elucidating the cryptic etiology of IBD would enable new preventative measures, diagnostics, and therapies.

Recent work has implicated the gut microbiome in the development and severity of IBD (10). Individuals afflicted with Crohn’s disease or ulcerative colitis, the two principal clinical forms of IBD, harbor taxa distinct from those present in healthy controls (11–14). Shotgun metagenomics further revealed that the abundances of several microbial metabolic pathways are significantly altered in IBD guts (13, 15, 16). These associations may be causal, because gut microbes can influence the immune system and intestinal homeostasis. For example, immunosuppressive regulatory T cells (Tregs) are prevalent in the colonic lamina propria (LP) compared to other organs. However, their numbers are reduced in germfree or antibiotic-treated mice, suggesting that microbiota affect colonic differentiation of peripheral Tregs (pTregs) (17, 18). A similar loss of Tregs occurs in people with polymorphisms in IBD susceptibility genes that promote defects in Treg responses (19). Thus, gut microbes have the potential to interact with immune cells and this interaction can be altered due to host genetics and other risk factors in the development of IBD.

We hypothesized that the changes in the immune status of individuals with IBD are associated with temporal alterations in the functional capabilities of their gut microbiota. Understanding how the gut microbiome dynamically changes during IBD and how these changes relate to host symptoms and immune activation could clarify which microbiomic alterations contribute to disease onset and progression and which alterations respond to disease. We are particularly interested in elucidating specific microbial pathways that may induce or exacerbate immune activation and in distinguishing these from pathways required for survival in an inflamed intestinal environment. Addressing these issues requires a prospective, longitudinal study of the microbiome in IBD.

Longitudinal investigations of the microbiome have tended to focus on taxonomic rather than functional changes (20, 21). One study used 16S sequencing in the T-bet^−/−^ RAG2^−/−^ ulcerative colitis (TRUC) mouse model of inflammatory disease to identify how gut microbiome taxonomic composition changes over the course of treatment-induced remission and then investigated how microbial pathway abundances might change over time with ancestral state reconstruction techniques (22). Shotgun metagenomic sequencing provides direct insight into the functions encoded in the microbiome, but it has not been applied to a longitudinal investigation of IBD. As a result, our insight into how the gut microbiome operates dynamically in association with disease development is limited.

Mouse models of disease present an opportunity to quantify the longitudinal covariation between gut microbiome functions and IBD development while overcoming the challenges associated with a prospective human study and reducing the extensive genetic, lifestyle, and microbiome variations among humans. We implemented this approach using a well-documented IBD model (23–29), where the activity of transforming growth factor β (TGF-β) dominant-negative receptor II is driven by the CD4 promoter CD4-dnTβRII (30) (called DNR here). TGF-β is important for inducing pTreg differentiation (31), and its signaling in naive T cells results in activation and nuclear translocation of Smad2/3 molecules and regulation of target genes, including Foxp3 (32–34). Foxp3 then provides a positive-feedback loop by downregulating Smad7, thereby reducing its inhibition of TGF-β signaling (35). Absence of TGF-β signaling in T cells results in loss of Foxp3 expression and defects in the *in vivo* expansion and immunosuppressive capacity of pTregs (36, 37). However, excess inflammation can also potently inhibit Foxp3 induction by TGF-β (38, 39), and the presence of certain inflammatory cytokines can instead divert differentiation of Tregs into pathogenic Th17 cells (40–45). Thus, due to the involvement of TGF-β in Treg cell differentiation and the requirement for Treg-produced interleukin-10 (IL-10) to maintain intestinal homeostasis, TGF-β signaling in T cells is an important component of intestinal immunity (46–54). Furthermore, mutations in both TGF-β and IL-10 signaling pathways have been implicated in human IBD (55–58). As a result of the blockage of TFGβ signaling on their T cells and of the reduced number of pTregs, DNR animals develop spontaneous colonic inflammation and IBD that is akin to Crohn’s disease (30, 59). In addition to these physiological similarities, the DNR line serves as an effective model of human IBD because (i) human IBD is associated with mutations in SMAD3 (5, 60–62), a direct downstream target of TGF-β RII required for Foxp3 induction in the gut (33), and (ii) DNR mice model the documented effect of Smad7 overexpression in human IBD (63–65).

To obtain insight into how the longitudinal dynamics of the microbiome associate with IBD onset and progression, we followed DNR and littermate wild-type (WT) controls from weaning through severe disease. We used shotgun metagenomics to quantify how fecal microbiome structure and function change over the course of disease development in DNR mice and identified components of the microbiome that both associate with and predict immune status. We focus on longitudinal changes in biological pathways (i.e., groups of genes performing a coherent function), using estimated abundances of KEGG modules from DNR and WT metagenomes. Our work indicates that the microbiome may contain biomarkers of IBD development, clarifies mechanisms through which the microbiome may contribute to disease development, and reveals how gut microbes operate to succeed in an inflamed intestinal environment.

## RESULTS

**FIG 1:**
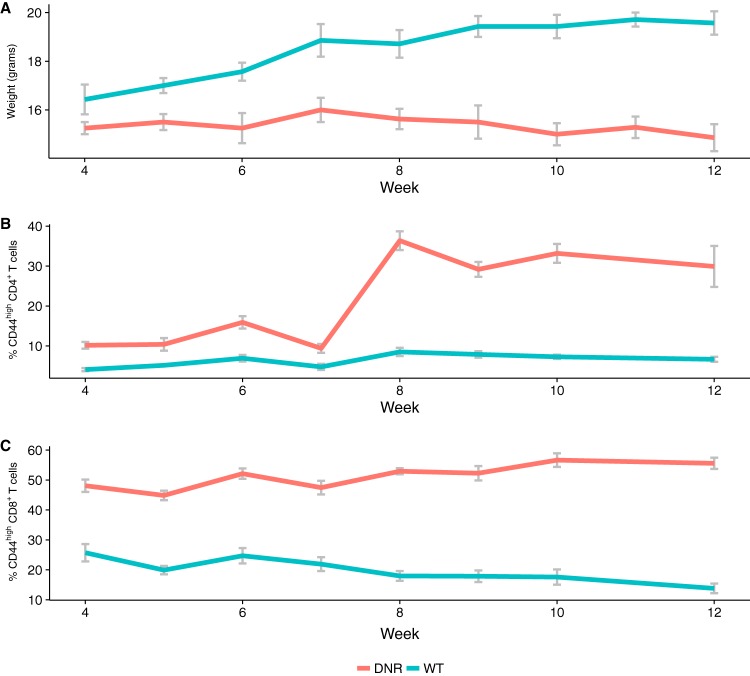
IBD development correlates with peripheral T cell activation in DNR mice. (A) Animal weight over time. *n* = 7 WT (blue) and 8 DNR (orange) mice. (B) Percentages of activated CD4 T cells among peripheral blood mononuclear cells (PBMCs). (C) Percentages of activated CD8 T cells among PBMCs. (B and C) *n* = 6 WT (blue) and 6 DNR (orange) mice. Error bars are standard errors of the means.

Age-matched female WT and DNR littermates were monitored longitudinally for IBD development over a period of 9 weeks, starting at 4 weeks of age, upon being weaned from their mother. As this is a T cell-mediated IBD model, we quantified peripheral CD4 and CD8 T cell activation by flow cytometry and measured the longitudinal change in the CD44^hi^ activated fraction, which includes both effector and memory T cells (see Fig. S1 in the supplemental material). We also measured the weight of the animals over time (Fig. 1A). As expected, WT mice gained weight and maintained a constant fraction of activated T cells. DNR mice, conversely, stopped gaining weight and experienced a sharp increase in CD4 T cell activation followed by a gradual increase in CD8 T cell activation starting at 7 weeks of age (Fig. 1). These results indicate that in our facility, the DNR mice developed signs of IBD starting around week 7 and full disease by week 9. DNR mice had to be euthanized by week 15, as they had lost more than 15% of their maximum body weight. Similar to the T cell activation phenotype observed in the blood after week 7 (Fig. 1B and C), the DNR animals had a larger fraction of activated T cells in the spleen and the gut-draining mesenteric lymph node (MLN) at week 15 (Fig. S2).

10.1128/mSystems.00036-17.2 FIG S1  Longitudinal phenotypic monitoring of T cell activation in PBMCs of WT and DNR “bleeder” mice. Representative FACS plots of data from WT bleeder no. 89 and DNR bleeder no. 85 determined at the indicated time points are shown. The gate is set for the CD44^hi^ population to show the percentages of activated effector and memory T cells over time. Download FIG S1, EPS file, 2 MB. Copyright © 2017 Sharpton et al. 2017 Sharpton et al. This content is distributed under the terms of the Creative Commons Attribution 4.0 International license.

10.1128/mSystems.00036-17.3 FIG S2  Phenotypic monitoring of CD8 T cell activation in WT and DNR “pooper” mice after disease onset. Representative FACS plots correspond to spleen or mesenteric lymph node (MLN) isolated from WT pooper no. 90 and DNR pooper no. 83 at week 15. The fractions of activated (CD44^hi^) and effector (CD44^hi^ KLRG-1^hi^) CD8^+^ T cells are shown. Download FIG S2, EPS file, 1 MB. Copyright © 2017 Sharpton et al. 2017 Sharpton et al. This content is distributed under the terms of the Creative Commons Attribution 4.0 International license.

We used shotgun metagenomics to assess how the functional potential of the gut microbiome diversifies over the course of disease progression. Specifically, we collected stool samples from parallel cohorts of DNR and WT mice weekly and performed shotgun metagenomic sequencing of samples obtained from the mice when they were 4, 5, 6, 8, 10, 12, and 13 weeks of age (see Table S1 in the supplemental material). We then quantified the abundance of KEGG modules encoded in each metagenome with ShotMAP (16), which revealed 373 modules present in at least one sample. These module abundances were then used to quantify how the within-sample diversities (alpha-diversities) of microbiome functions differed over time in DNR and WT mice. A. Kruskal-Wallis test performed to analyze the change in KEGG module Shannon entropy over time (Fig. S3) found that the DNR mice were relatively stable in their functional alpha-diversity (*P* = 0.47) compared to WT mice (*P* = 0.078). We also observed that functional alpha-diversities differed among individuals within a line over time and that this variation differed between lines in association with disease activation (week 7). Specifically, the coefficient of variation (CV) of KEGG module Shannon entropy data from a generalized linear model was higher among WT mice than DNR mice after disease activation (*P* = 0.0085). We also found that the CV was higher among DNR mice prior to activation, though this difference is reduced when the disproportionately variable week 5 samples are removed from the analysis (*P* = 0.21). These results show that the functional diversity of the mouse gut microbiome is relatively constrained early in life but increases over the lifetimes of WT but not DNR individuals.

10.1128/mSystems.00036-17.4 FIG S3  Levels of functional alpha-diversity of the gut microbiome as measured by Shannon entropy differed over time across cohorts (left). In fact, there was significantly greater variation in the levels of Shannon entropy between the lines corresponding to the periods before and after disease onset (right). Download FIG S3, EPS file, 1.5 MB. Copyright © 2017 Sharpton et al. 2017 Sharpton et al. This content is distributed under the terms of the Creative Commons Attribution 4.0 International license.

10.1128/mSystems.00036-17.7 TABLE S1  Project metadata. Download TABLE S1, XLSX file, 0.02 MB. Copyright © 2017 Sharpton et al. 2017 Sharpton et al. This content is distributed under the terms of the Creative Commons Attribution 4.0 International license.

**FIG 2:**
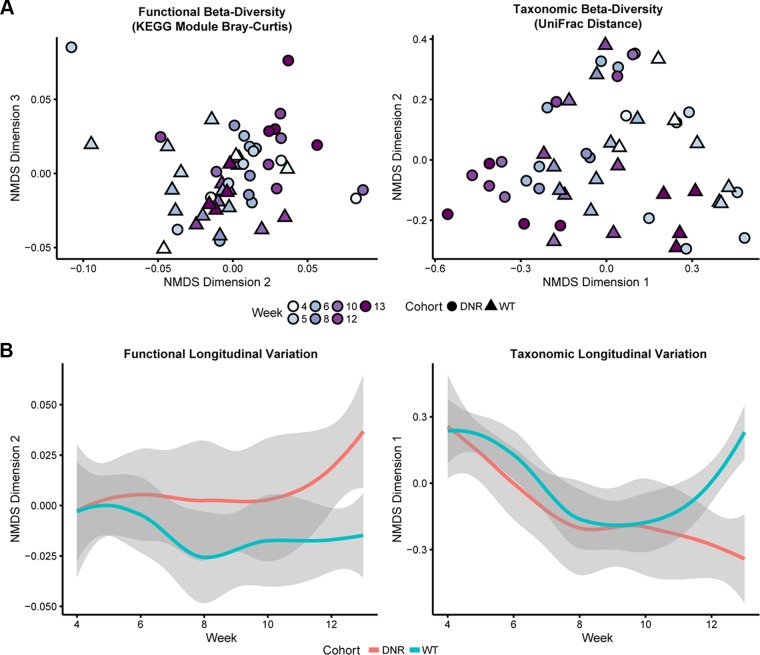
The taxonomic and functional diversity of the gut microbiome associates with IBD development. (A) NMDS ordination plots of the functional (left) and taxonomic (right) beta-diversity of samples from each line illustrate the significant divergence in levels of beta-diversity between lines over time. Functional beta-diversity was measured as Bray-Curtis dissimilarity based on KEGG module abundances, while taxonomic beta-diversity values represent the UniFrac distances of taxa detected in metagenomes. (B) The longitudinal variation of samples along selected NMDS dimensions similarly reveals how DNR and WT lines significantly diverge over time in terms of both their functional (left) and their taxonomic (right) beta-diversity. Smoothed (locally weighted scatterplot smoothing [LOESS]) trajectories of samples from each line over time are plotted, where gray areas represent 95% confidence intervals.

We then investigated how the composition of gut microbiome functions varies over time and between cohorts (DNR mice versus WT mice) by using an abundance-weighted beta-diversity metric (Bray-Curtis dissimilarity). At a global level, KEGG module abundances were similar in DNR and WT mice prior to week 6 but then diverged over time as IBD developed in the DNR mice (Fig. 2). Furthermore, the diversity of KEGG modules found in a metagenome was significantly associated with the week that the sample was collected within the cohort (permutational multivariate analysis of variance [PERMANOVA] *P* = 0.01, *R*^2^ = 0.42), as well as with the cohort’s weekly mean activated T cell status (pcCD4tCD44hi) (PERMANOVA *P* = 0.001, *R*^2^ = 0.16). Thus, there exist microbiome-encoded functional modules that differ in abundance in association with IBD progression in DNR mice.

This temporal divergence in DNR versus WT microbiome functions was mirrored in the taxonomic structure of the microbiome (Fig. 2). The compositions of the gut metagenomes of the WT and DNR lines were relatively similar at early time points and began to diverge at week 6. Additionally, the microbiomes of WT mice remained relatively consistent over time compared to those of DNR mice, though they were not without temporal variation. Indeed, similarly to the functional diversity analysis, the levels of taxonomic beta-diversity of the microbiome significantly differed between the lines over time (PERMANOVA *P* = 0.004, *R*^2^ = 0.46), though not in a manner that corresponded to mean activated T cell status (PERMANOVA *P* = 0.118, *R*^2^ = 0.046). Collectively, these analyses indicate that (i) the diversities and structures of the gut microbiome differ over time in WT and DNR mice that are between 4 and 15 weeks of age; (ii) the WT and DNR microbiomes are generally consistent with each other prior to immune activation in DNR mice but diverge afterward; and (iii) immune activation is associated with changes in the subsequent successional diversification of the gut microbiome.

On the basis of these observations, we assessed how specific components of the microbiome associate with disease development. A key novelty of our approach is the use of Tweedie compound Poisson generalized linear mixed-effects models (GLMMs). These models allow us to test for differences in temporal trends in KEGG module abundance between DNR and WT mice while accounting for baseline differences between mice and genotypes, as well as for DNA extraction kit effects. GLMMs enable accurate modeling of non-normally distributed abundance data and correctly account for multiple sources of variation (66), including the intersubject variation that is present in repeated-measures designs such as the longitudinal sampling of individual mice in our study. The Tweedie compound Poisson distribution, which represents a weighted mixture of Poisson and gamma distributions, has a number of other attractive features. Its exponential relationship between variance and mean captures the overdispersion that is frequently present in environmental DNA sequence data, and its point mass at zero allows one-step fitting of zero-inflated data (versus fitting a model to determine the presence or absence of a feature before modeling nonzero components, as in hurdle models). Additionally, the Tweedie compound Poisson distribution is a continuous distribution, allowing us to use a normalized abundance measure, instead of raw counts, as the dependent variable. We provide a more detailed description of the models used in our analysis in Text S1 in the supplemental material.

10.1128/mSystems.00036-17.1 TEXT S1  Modeling methods. Download TEXT S1, DOCX file, 0.1 MB. Copyright © 2017 Sharpton et al. 2017 Sharpton et al. This content is distributed under the terms of the Creative Commons Attribution 4.0 International license.

**FIG 3:**
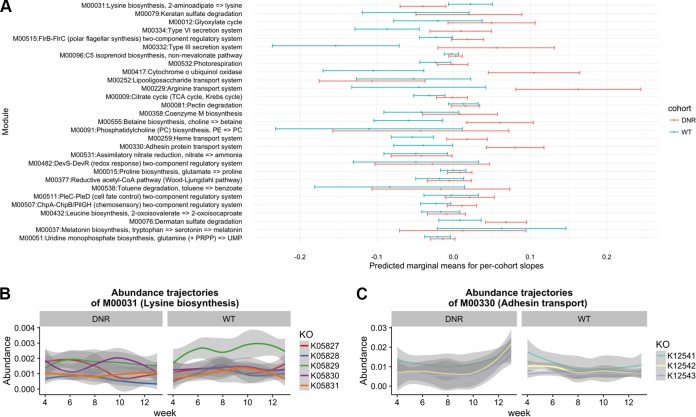
Summary of GLMM results from 29 modules with significant time by group interaction. (A) The quantity plotted is the predicted marginal mean (PMM) of the slope coefficients. Significance testing was done by comparing goodness-of-fit values from full and reduced GLMM specifications, and the full model was used to produce the PMM estimates shown here. This quantity was primarily calculated to get a succinct summary of the direction of temporal change and does not always coincide with the interaction coefficient that is the focus of the main analysis. The estimates were obtained by running the lstrends function from the lsmeans R package (134). (B) The underlying KO abundance trajectories of a significant module (M00031; lysine biosynthesis) that decreases in abundance in DNR mice and increases in abundance in WT mice over time, as evidenced by a negative model slope and a positive model slope, respectively. (C) The plot was constructed as described for panel B, except that this significant module (M00330; adhesin transport) significantly increased in abundance over time in DNR mice whereas it did not change in abundance in WT mice. For both panel B and panel C, the shaded ribbons represent LOESS confidence bounds.

We first looked at overall trends of abundance trajectories for DNR mice versus WT mice as quantified by the interaction between genotype and time in the GLMM. These analyses revealed 29 KEGG modules with significant differences in abundance trends between DNR and WT mice (false-discovery rate [FDR] < 0.05). The interaction coefficient was positive for 26 of the significant modules (Table S2), which indicates that those modules became increasingly abundant in DNR mice versus WT mice over time. This set includes modules associated with UMP biosynthesis (M00051), keratin sulfate degradation (M00079), and the type III secretion system (M00332). The three modules with negative interaction coefficients, indicating decreasing abundance in DNR mice versus WT mice over time (Fig. 3), are lysine biosynthesis (M00031), lipooligosaccharide transport (M00252), and melatonin biosynthesis (M00037).

10.1128/mSystems.00036-17.8 TABLE S2  KEGG modules that exhibit significant group by time interaction coefficients, indicating that they differentially diversified between the two lines over time. Download TABLE S2, XLSX file, 0.1 MB. Copyright © 2017 Sharpton et al. 2017 Sharpton et al. This content is distributed under the terms of the Creative Commons Attribution 4.0 International license.

**FIG 4:**
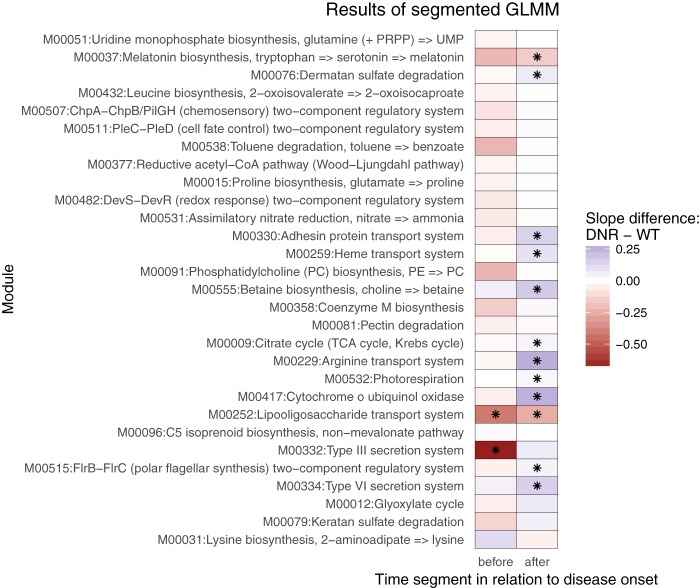
Modules with slopes significantly differing between groups showed primarily post-disease-onset differences in analyses performed with a segmented GLMM. For each cohort, the segmented GLMM estimate data represent two separate WT slopes (pre-week 7 and post-week 7) and two deviations from those slopes, which represent the time by group interaction that measures how DNR slopes differ from WT slopes. The estimates of these deviations are plotted, with asterisks marking coefficients that were significantly nonzero, with B-H-corrected *P* values of <0.2.

To obtain improved temporal resolution regarding the divergence of module abundance in DNR mice, we extended our GLMMs to include a “hinge” at week 7, which is when immune activation initiates in DNR mice. This segmented regression approach has the potential to reveal modules that diverge in abundance between DNR and WT mice either between weeks 4 and 7 or between weeks 7 and 13. Only 13 of the 29 previously identified modules exhibited a significant effect using segmented regression (Fig. 4), likely due to a loss of power from partitioning the data into two smaller sets of samples. However, for these 13 modules, our results clarify when the DNR and WT abundances began to diverge (Table S3). The predominant pattern consisted of similar module abundances prior to week 7, followed by divergence after immune activation (11/13 modules). This pattern suggests that these modules respond to disease or play a role in disease progression.

10.1128/mSystems.00036-17.9 TABLE S3  KEGG modules with significant interactions in the segmented GLMM analysis. Download TABLE S3, XLSX file, 0.03 MB. Copyright © 2017 Sharpton et al. 2017 Sharpton et al. This content is distributed under the terms of the Creative Commons Attribution 4.0 International license.

Lipooligosaccharide transport (M00252), which is a two-component system with an unknown substrate in the mammalian gut, was the only module that stratified DNR and WT mice both before and after disease onset. To further investigate the potential taxa that may drive this particular signal, we assessed the taxonomic source of the KEGG sequences that recruited metagenomic reads into the module. We also quantified the distance covariance (67) between the longitudinal trajectories of the KEGG orthology groups (KOs) that comprise the module and the trajectory seen with each observed species. The results were mixed, with the former analysis suggesting the presence of primarily *Streptococcus* contributions, while the latter identified the greatest similarity with *Lactobacillus murinus* and "*Candidatus* Arthromitus" group trajectories (Fig. S4). The differences in the taxonomic composition of the reference data underlying these two approaches could account for these inconsistencies, as could the fact that the KEGG analysis relies on amino acid comparisons whereas the species trajectories are determined through nucleotide comparisons. Thus, an uncharacterized lipooligosaccharide transporter encoded in *Streptococcus* and other gut microbes decreases in abundance over time at a significantly higher rate in DNR mice than in WT mice, starting early in life before weight loss and immune activation.

10.1128/mSystems.00036-17.5 FIG S4  Two different analyses potentially explain the taxonomic origins of the lipooligosaccharide transport KOs that were observed. (A) Species identities of KEGG ortholog sequences that recruited reads in generating the relevant KO abundances. (B) Values corresponding to distance covariance (dCov) trajectories of K09694 and K09694 and all species, with asterisks marking those dCov values that were significantly nonzero after B-H multiple-testing correction. Download FIG S4, EPS file, 2 MB. Copyright © 2017 Sharpton et al. 2017 Sharpton et al. This content is distributed under the terms of the Creative Commons Attribution 4.0 International license.

The temporal changes seen with the type III secretion system (M00332) differed between the lines uniquely before disease onset. Specifically, the module decreased in abundance in WT mice over weeks 1 to 7, with KO K03225 primarily driving this effect. On the other hand, this module was relatively stable in DNR mice prior to disease onset, and several of the KOs that comprise the module increased in abundance in DNR mice in the later weeks (Fig. 4). The discovery of a stable, rather than decreasing, abundance of K03225 as an early indicator of IBD in DNR mice is intriguing because type III secretion systems are used by pathogens to invade the gut community and alter the gut environment (68, 69).

We next examined baseline differences in module abundance between DNR and WT mice at weaning. Early differences could result from genotype-specific selection of the gut microbiome or cage effects. Our models revealed 17 modules with significantly different intercepts (FDR < 0.05), which indicates differences in the abundances of the two lines at week 4 (Table S4). Eight of these modules, including several methanogenesis-associated pathways, had positive intercept coefficients, meaning that they were more abundant in DNR mice than in WT mice at week 4. Lipopolysaccharide biosynthesis and eight other modules showed the opposite effect and were higher in abundance in WT mice at weaning. This early-life variation in the microbiome supports hypotheses that suggest that preadolescent development of the microbiome can affect health state later in life. However, these temporal relationships are complex; later changes in abundance, as captured by the time by cohort interaction (which measures the difference in slopes between DNR and WT mice), could reverse the pattern seen at weaning.

10.1128/mSystems.00036-17.10 TABLE S4  KEGG modules with significant intercepts, indicating that they exhibited significantly different abundances between lines at the initial time point. Download TABLE S4, XLSX file, 0.04 MB. Copyright © 2017 Sharpton et al. 2017 Sharpton et al. This content is distributed under the terms of the Creative Commons Attribution 4.0 International license.

**FIG 5:**
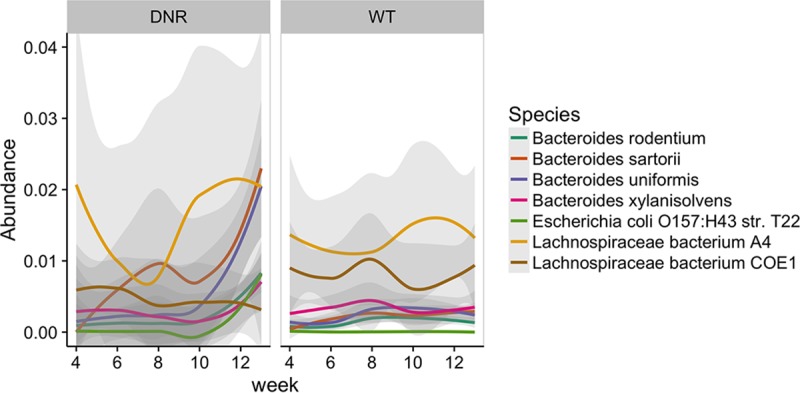
Species that showed significantly different trajectory shapes between DNR and WT groups. These results are based on an FPCA-based goodness-of-fit comparison test that identified 7 species that were different at an FDR of <0.05.

To explore the temporal dynamics of specific gut taxa, we applied the same GLMM analysis to species abundances. This analysis yielded no significant results at an FDR of <0.05, likely due to not having the advantage of grouping components across a higher-order variable. While species could be grouped into higher taxonomic entities, the model assumption that members of the same group tend to covary across samples and over time may be violated because members of the same taxonomy may compete with or ecologically exclude one another (70). We evaluated this possibility by applying nonparametric decomposition of variance components (71) to assess whether within-module or within-genus dispersion decomposition patterns were significantly different from those obtained from random permutations of the underlying data. This auxiliary analysis found that components of functional groups covaried more than components of randomly chosen groups whereas components of taxonomic groups did not (Fig. S5). This observation indicates that grouping taxa would violate GLMM assumptions. Consequently, we instead used a goodness-of-fit test based on functional principal-component analysis (FPCA), which is less rigid in its assumption of linearity and capable of borrowing information across species due to the representation of abundance trajectories as combinations of eigenfunctions derived from the entire data set. This test identified seven species that significantly differed in their levels of variation over time between the DNR and WT cohorts (Fig. 5; Table 1), including greater increases in abundance over time within DNR microbiomes for *Escherichia coli* and four species from the *Bacteroides* genus, which are associated with gut inflammation (10).

10.1128/mSystems.00036-17.6 FIG S5  Distributions of F-statistics computed by the DISCO method for KO and species vectors within module and genus groupings, respectively, compared to values from permuted groupings. Results of Kolmogorov-Smirnov tests show significant differences between real and permuted distributions in the functional groupings but no significant differences in the taxonomic groupings. Download FIG S5, EPS file, 1 MB. Copyright © 2017 Sharpton et al. 2017 Sharpton et al. This content is distributed under the terms of the Creative Commons Attribution 4.0 International license.

**TABLE 1:** Species with significantly different trajectory shapes in the FPCA-based goodness-of-fit comparisons^a^

Species ID	*P* value	FDR	Species name	WT area under LOESS curve	DNR area under LOESS curve
54642	0	0	*Bacteroides sartorii*	0.01992	0.07699
57185	0	0	*Bacteroides xylanisolvens*	0.03051	0.02506
57318	0	0	*Bacteroides uniformis*	0.02297	0.04506
58110	0	0	*Escherichia coli* O157:H43 strain T22	5.35E−4	0.007523
59684	0.0001	0.0025	*Lachnospiraceae* bacterium COE1	0.07203	0.04213
59708	0	0	*Bacteroides rodentium*	0.0136	0.01986
61442	0.0013	0.02786	*Lachnospiraceae* bacterium A4	0.119	0.1348

a LOESS, locally weighted scatterplot smoothing.

## DISCUSSION

The results of this study represent the first shotgun metagenomic characterization of IBD development. By using a controlled mouse model, a longitudinal study design, and statistical modeling, we identified novel microbial biomarkers associated with IBD onset and progression. Many of the taxa and functions that we implicated have known roles in immune regulation and pathogenicity, making them plausible candidates for stimulating the disease process, while others likely represent responses of the microbiota to changes in host physiology. Ordination and GLMM analyses enabled us to distinguish these scenarios by identifying significant differences between DNR and WT mice over time (from weaning through severe disease). We discovered that the lipooligosaccharide transport and type III secretion protein abundance trajectories that occur between weaning and immune activation differentiate DNR mice prior to immune activation, making them promising early biomarkers and consistent with a potentially causal role in IBD. Abundances of 17 modules are altered in DNR mice at weaning and could predict IBD risk if they generalize to other mouse models and human disease (see below). Many other modules as well as a few species have altered abundances in DNR mice in later, more-severe stages of disease. Functional and taxonomic diversities also show temporal differences in DNR mice that correlate with immune profiles and/or disease progression. Most of these discoveries would have been missed in a cross-sectional study because the disease association is a longitudinal trend.

By using shotgun metagenomics, we were able to investigate both taxonomic and functional characteristics of the IBD microbiome. Both types of data consistently showed differences between DNR and WT mice. For example, beta-diversity analyses revealed increasing divergence of both taxonomic and functional profiles between DNR and WT microbiomes over the last 4 weeks of the study. In addition, the individual taxa and modules with genotype-specific trajectories predominantly had increased abundance in DNR mice after disease onset. These similarities in the successional diversification of species and genes support the idea that taxonomic changes in IBD have functional consequences that are linked to immune activation. Despite such parallels, our taxonomic and functional results differed in several important ways. Notably, the number of species that stratified lines over time was lower than the number seen with KEGG modules. Furthermore, most of the IBD-associated modules that we discovered were not represented solely in singular species and would have been missed by considering information from taxonomic analyses only. These results could have been due to disease-associated functional redundancy, wherein a gene that is enriched in DNRs might derive from a different species in each mouse. Other potential reasons include (i) higher power due to grouping protein families into modules and (ii) missed taxonomic associations due to the relatively low number of laboratory mouse-associated microbes in the genome tree of life (72). Future work should explore how taxa missed by reference-based quantitation vary in association with IBD in DNR mice.

Despite finding relatively few species that distinguish DNR mice, we can gain insight into the disease process from what is known about how these taxa interact with the host. It is striking that four of the seven species that change in abundance as IBD develops belong to the genus *Bacteroides* and that three of them are more abundant in DNR mice. Several studies have implicated *Bacteroides* in intestinal inflammation. For example, the members of a subset of *B. fragilis* strains carry a proinflammatory metalloprotease toxin that has been identified in 19.3% of patients with active IBD (73), and the inoculation of animals with such strains is associated with severe colitis (10, 74). Subsequent research showed that multiple commensal species of *Bacteroides* could be incorporated into the gut microbiomes of IBD-susceptible genotypes of mice, including mice with TGF-β susceptibility loci, to induce IBD (75). Supporting the idea that *Bacteroides* species contribute to IBD, we observed a modest increase (FDR = 0.1898) in the hemophore/metalloprotease transport system module (M00328) in DNR mice as disease progressed. These and other mechanistic hypotheses must be tested, because the species of *Bacteroides* that we identified are diverse and species within the same genus can exhibit discordant patterns of interaction with host physiology (76).

Cross-sectional and mechanistic investigations of IBD support our finding that disease development is linked to microbiome taxonomy and function (4, 77, 78). The occurrence of progressive divergence of DNR and WT microbiomes as IBD worsens is consistent with a 16S-based study using a different mouse model of IBD in which gut microbes and imputed functions changed in association with disease status and therapeutically induced remission (22). Additionally, studies in germfree mouse models of IBD implicate the gut microbiome in disease development. For example, interleukin-10 (IL-10) knockout mice grown under germfree conditions do not develop colitis, whereas conventionally raised mice do (79). Similar findings have been reported for the TRUC mouse model (80). Furthermore, IL-10 knockout (81) and IL-2-deficient (82) mice manifest differential levels of severity of colitis dependent on the types of taxa that colonize their gut. Human studies of IBD have yet to investigate the disease longitudinally. However, our results are consistent with microbiome case-control studies that found significant differences between the taxonomic (11, 83–87) and functional (13, 16, 22) profiles of IBD patients and those of healthy controls, especially in cases of Crohn’s disease. Additionally, clinical administration of antibiotics shows promise for reducing the intestinal inflammation associated with IBD (88, 89). The longitudinal biomarkers that we identified are promising new candidates for investigation in the context of human disease onset and progression.

Our analyses identified several modules that implicate a pathogenic effect by the DNR microbiome. For example, DNR mouse microbiomes show increases in the abundance of adhesion protein transport modules (M00330) in association with disease, which may help pathobiotic members of the microbiome associate with and metabolize intestinal mucosa (90). Correspondingly, keratan (M00079) and dermatan (M00076) sulfate degradation pathways increase in abundance as disease progresses. Keratan sulfate and dermatan sulfate are glycosaminoglycans (GAGs) that are integral to intestinal mucosa and regulate the permeability of the gut epithelium. These sulfated GAGs are depleted in IBD patients (91), and their metabolism by intestinal bacteria, including *Bacteroides thetaiotaomicron*, contributes to intestinal colonization (92, 93). Furthermore, Crohn’s metagenomes exhibit an increase in abundance in GAG degradation pathways (16). DNR guts also have elevated levels of type III and type IV secretion systems, which pathogenic organisms leverage to successfully invade the gut microbiome and induce preferable ecological conditions within the gut (68, 69). Curiously, type III secretion abundance shows the opposite effect before immune activation (weeks 4 to 7), perhaps because of broad shifts in community composition after week 7 or alternatively due to microbes with type III secretion systems invading the LP and becoming less abundant in stool over time. Finally, we observed an increase in modules associated with the biosynthesis of isoprenoids, which have been linked to the stimulation of the mammalian immune system (94). Together, these DNR-associated pathways support a pathogenic role of gut microbes in IBD development. Future studies that seek to determine the existence of a microbiome-mediated etiology for IBD should consider these potential mechanisms of disease activation.

Our identification of pathways that change in association with IBD development generates many novel hypotheses about the mechanisms through which gut microbes contribute or respond to disease development. Future studies can explicitly test these hypotheses to discern the cause-and-effect relationship between the gut microbiome and inflammatory bowel disease. Several KEGG modules with different abundance dynamics in DNR mice versus WT mice appear to be associated with the microbiome’s acclimation to the disease environment. For example, we observed increases in the abundance of two-component systems (M00511, M00482) that may contribute to a cell’s ability to manage the elevated oxidative stress that exists during active IBD (95). We also observed increases in abundance in pathways associated with cellular chemotaxis (M00515, M00507). This result is consistent with observations of increased cell motility pathways in the gut microbiomes of TRUC mice suffering active colitis using imputations from 16S data (22). This result also aligns with prior work that implicated Toll-like receptor recognition of flagellar bacterial antigens in the development of intestinal inflammation (96, 97). On the basis of these observations, we speculate that, given that intestinal permeability frequently increases during IBD flare-ups, chemotaxis pathways help microbiota scavenge the metabolic resources required to survive inside an inflamed gut or to invade the host (98).

We also observed several biosynthetic modules that increased in abundance in association with IBD development. For example, the modules related to the biosynthesis of UMP, leucine, proline, and ammonia changed in association with disease. These results may suggest that the metabolic preferences and needs of the organisms that comprise the microbiome change as disease develops. Alternatively, it may be that more T cells are entering the gut, becoming activated, and consequently consuming the local resources, which in turn results in bacteria activating biosynthetic pathways to survive and compete. Our finding that pathways associated with ammonia production (M00531) increase in abundance in DNR mice is noteworthy because prior studies have found that IBD associates with a lower pH in the intestinal lumen (99), and the production of ammonia by bacteria may serve to buffer such pH changes. Additionally, these pathways are utilized when bacteria metabolize proteins, amino acids, and urea, and the increase in this pathway may indicate a preferential utilization of these substrates by the microbiome (or, as described above, immune cells) during disease.

Furthermore, we observed increases in modules associated with choline metabolism, specifically, in betaine and phosphatidylcholine biosynthesis modules. Recent work has connected the gut microbiome’s production of these metabolites to increased cardiovascular disease risk (100). Our finding is important because a growing number of studies indicate that IBD patients have an increased risk of developing cardiovascular disease, especially during flare-ups (101, 102). The mechanisms underlying this increased risk are not well resolved but may relate to a proposed explanation for the increased cardiovascular disease risk observed in HIV-infected patients (103, 104). In this model, changes in the relative proportions of protective and pathobiotic gut microbiota, especially those capable of translocating across the gut epithelium, activate a chronic systemic inflammation that increases cardiovascular disease risk. It is thus tempting to speculate that, on the basis of our observations in these mouse models of disease, IBD-associated and perhaps HIV-associated changes in the microbial metabolism of choline contribute to or at least indicate the presence of this increased risk of cardiovascular disease.

Another intriguing hypothesis emerges from our observation that levels of heme transport genes are elevated in DNR mice as IBD develops. Bacteria use this module to scavenge iron from the environment. Iron is a crucial component for many cellular processes, but gut microbes seldom have access to free iron and instead sequester it from host sources, such as heme (105, 106). Heme concentrations may be increased in IBD, as a common feature of the disease is intestinal bleeding (107). Hence, we hypothesize that gut microbes that can take advantage of this heme may flourish in DNR mice. It is intriguing to further speculate that microbial sequestration of heme contributes to IBD (e.g., through signaling to the immune system) or to iron deficiency in IBD patients (108).

One surprising discovery was an increase in the abundance of pathways associated with the production of benzoate (M00538) in DNR mice. Benzoate is a carboxylic acid produced by microbial degradation of dietary aromatic compounds and is a precursor of hippurate biosynthesis in mammals (109). Prior work suggested that hippurate may be a useful diagnostic of Crohn’s disease given that it is found at significantly lower levels in the urine of patients (109) and that the gut microbiome’s production of benzoate is responsible for these differences in urinary hippurate levels (110). Our results are inconsistent with this prior work in that they indicate that intestinal benzoate biosynthesis levels are higher in sick animals. This difference may be due to variations in the host species being investigated, including how benzoate is subsequently metabolized in the gut or by the host. Alternatively, the potential of the DNR microbiome to make excess hippurate may not be realized given that we performed DNA sequencing. Future mechanistic studies could measure benzoate and hippurate levels and quantify the benzoate proteins at the RNA or protein level in DNR mice versus WT mice.

Most of the taxonomic and functional IBD biomarkers that we identified become increasingly abundant in DNR mice throughout the disease process. But three modules, namely, melatonin biosynthesis (M00037), lysine biosynthesis (M00031), and lipooligosaccharide transport (M00252), show the opposite trajectory and decrease in abundance over time in DNR mice relative to WT mice. Melatonin has a dual effect on the immune system, acting in a stimulatory manner in early infection and in an immunomodulatory manner in cases of prolonged inflammation (111). The effects of melatonin produced by gut commensals have not been studied as extensively as those of endogenous melatonin. Traditionally, melatonin acts as a potent antioxidant, although additional quorum signaling functions in bacteria have been recently reported (112). The reduction in melatonin biosynthesis capacity observed in the DNR mice could have been caused by the expansion of species that can tolerate a highly oxidative environment (113) or by microbes that utilize other strategies for neutralizing reactive oxygen species. Without metabolite data, it is not possible to definitively say that the final concentrations of melatonin are reduced in the disease state, since the decrease can be offset by host production. With respect to lysine biosynthesis, this module is also depleted in human IBD microbiomes (13), indicating that there may exist similar mechanisms of interaction between disease context and the gut microbiome across species. Future work should empirically test the potential role of these microbiome functions in the development of IBD, especially in individuals that are genetically susceptible to the disease.

Lipooligosaccharide transport is the only module to show significant differences in abundance trajectories both preactivation and postactivation. Intriguingly, its abundance was consistently lower in DNR mice than in WT mice throughout our study, with the largest difference occurring during weeks 4 to 7, prior to immune activation and the manifestation of disease symptoms. This finding seems surprising initially, because lipooligosaccharides are the major glycolipids that are produced by mucosal Gram-negative bacteria and are known to have proinflammatory effects (114). However, the two genes (NodI and NodJ) in the lipooligosaccharide transport system are present across diverse prokaryotes, and the substrates of this two-component ABC transporter have not been characterized beyond lipochitin oligosaccharide export in rhizobial bacteria (115, 116). Determining what this system transports in the mammalian gut and how its function changes in IBD is an exciting prospect. Regardless of the mechanism, the consistent and presymptomatic depletion of lipooligosaccharide transport genes in DNR mice makes this module a promising candidate biomarker for predicting and diagnosing IBD.

We relied on a mouse model to quantify the longitudinal interaction between the gut microbiome and disease because the extensive interindividual variations in human genetics, lifestyle, microbiome composition, and disease status and severity can complicate study design, analysis, and interpretation. We used the DNR mouse model because it is relevant to our understanding of the mucosal immunological dysregulation that occurs during human IBD and, consequently, of its interaction with the gut microbiome. Indeed, we observed immune activation in the blood of the DNR mice that was consistent with what has been observed in human IBD (117). The phenotype observed in DNR mice is akin to severe Crohn’s disease, with relatively substantial immunological activation and weight loss by week 12. Interpretations of the microbiome-disease interaction in this model should take into consideration this relatively severe disease status. Alternative mouse lines may be better models for other forms of IBD. Another consideration is that we found some baseline differences in microbiome protein abundances in DNR mice at weaning that may be specific to this genetic model of IBD. Ultimately, comparisons between our results and those obtained by the integrated Human Microbiome Project (iHMP) (118), which is longitudinally evaluating the microbiome and immune status of IBD patients, will clarify the relevance of the findings produced by the DNR model to human populations. Additionally, future research should use this model and build upon our findings to clarify how TGF-β-induced differentiation and function of T cells interact with the taxonomic structure and function of the gut microbiome.

Overall, our results indicate that the development of IBD is associated with corresponding changes in the operation of the gut microbiome. Microbial taxa and KEGG module abundances vary over time and in association with immune activation. Furthermore, our results suggest that the gut microbiome may contribute to disease by activating inflammation through metabolism of mucosa and by expressing proinflammatory and downregulating anti-inflammatory metabolites. Because our study relied on the imputation of microbiome function from DNA sequences, we cannot definitively conclude that the observed differences in the microbiome’s functional profiles manifest as differences in the metabolites produced by the microbiome. Future research that applies direct measurements of microbiome function should be used to validate and expand the results presented here. Regardless, our results hold promise for our understanding of microbiome-mediated IBD disease mechanisms and the potential of using microbiome sequencing of patient stool to classify and potentially even predict disease.

## MATERIALS AND METHODS

### Growth of mice and microbiome sampling.

We bred two cohorts of DNR and WT littermate control animals in the Gladstone Institutes mouse facility as follows. CD4-dnTβRII (DNR) animals were crossed to the RAG1^−/−^ background to eliminate the T cell-mediated IBD and were transferred from Yale University to Gladstone Institutes in 2010. To initiate the experiments described in this study, DNR-RAG1^−/−^ males were bred with C57BL/6N female animals, and DNR-RAG1^−/+^ progeny were again crossed to C57BL/6N females to generate a combination of RAG1^−/+^ and RAG1^+/+^ DNR and WT age-matched littermate controls. Animals were given regular chow consisting of irradiated PicoLab Rodent diet 20 (LabDiet). Only female animals were used in this study. Four cohoused WT-RAG1^+/+^ and five cohoused DNR-RAG1^+/+^ littermates were followed longitudinally for 15 weeks, and fresh fecal samples were collected weekly and stored at −30°C until they were subjected to microbiome processing. All mice from both cohorts were weighed weekly. All animal experiments were conducted in accordance with guidelines set by the Institutional Animal Care and Use Committee of the University of California, San Francisco.

### Immune sampling.

Tail vein blood samples were collected weekly from a parallel cohort of “bleeder” mice (*n* = 6 WT, *n* = 6 DNR) to quantify how their immune status changed over time. These were distinct individuals from the “pooper” mice cohort (same colony and time period) subjected to stool metagenomics in order to prevent repeated tail vein blood sampling from affecting the health or microbiota of the cohort of pooper mice. Specifically, ∼100 μl (2 to 3 drops) of blood from tail vein was added to 30 μl of 1× heparin (500 units/ml). A 500-μl volume of 1× ACK (ammonium-chloride-potassium) lysis buffer (Lonza) was added directly to the cells, and the mixture was incubated at room temperature for 2 to 3 min. Cells were centrifuged at 4,000 rpm for 5 min. The top layer was aspirated, and another 500 μl of 1× ACK lysis buffer was added followed by centrifugation. Cells were resuspended in fluorescence-activated cell sorter (FACS) buffer (phosphate-buffered saline [PBS]–0.5% fetal bovine serum [FBS]), and, after blocking was performed, Fc receptors with anti-CD16/CD32, single-cell suspensions were incubated with fluorescein isothiocyanate (FITC) CD4 (GK1.5), phycoerythrin (PE) CD62L (MEL14), peridinin chlorophyll protein (PerCP)-Cy5.5 CD8a (53.6.72), and allophycocyanin (APC) CD44 (IM7) mouse antibodies for 30 min at 4°C. Stained cells were washed and acquired on an Accuri C6 cytometer (BD). Blood lymphocytes were gated on CD4^+^ or CD8^+^ fractions, and percentages of activated/memory (CD44hi) cells among CD4^+^ and CD8^+^ T cells were determined using FlowJo software (Tree Star Inc.). This cohort was separated from those subjected to microbiome sampling to eliminate the effect that repeated bloodletting might have on the microbiome. At 15 weeks of age, two WT mice and three DNR mice from the group of nonbleeding animals were euthanized. Spleen and mesenteric lymph nodes were then processed into single-cell suspensions and subjected to ACK lysis and cell surface staining as described for peripheral blood mononuclear cells (PBMCs). The level of T cell activation was quantified and found to highly correlate with the blood immune status of their “bleeder” littermates (see Fig. S2 and Table S1 in the supplemental material).

### Metagenome sequencing and analysis.

QIAamp DNA stool minikits (Qiagen, Valencia, CA) were used to extract DNA from stool samples collected at weeks 4, 6, 8, 10, and 12. Samples were incubated in a water bath at an elevated temperature of 95 C to increase the lysis of bacterial cells per manufacturer instructions. A MoBio PowerFecal DNA isolation kit (MoBio, Carlsbad, CA, USA) was used per manufacturer instructions to process stool samples collected at weeks 5 and 13. The kit type was accounted for in statistical modeling to adjust for any potential differences in extraction bias between the two methods.

Purified DNA was prepared for shotgun metagenomic sequencing using the Nextera XT library preparation method (Illumina, San Diego, CA, USA). Libraries were quality assessed using quantitative PCR (qPCR) and a Bioanalyzer (Agilent Technologies, Palo Alto, CA, USA) and subsequently sequenced using an Illumina HiSeq 2000 sequencing system. This produced an average of 74,427,303 100-bp paired-end sequences per sample. Metagenomic reads were quality controlled using the standard operating procedure defined by the Human Microbiome Project Consortium (119) as implemented in shotcleaner (120). Briefly, reads were quality trimmed using prinseq (121) and mapped against the mouse reference genome sequence (GRCm38) using bmtagger (122). Exact-duplicate reads were collapsed, and the subsequent high-quality data were subject to taxonomic and functional annotation. Functional annotation of metagenomes was conducted using ShotMAP as described in reference 16, with Prodigal (123) to call genes and RAPsearch2 (124) to identify metagenomic homologs of the KEGG database (downloaded February 2015). Reads mapping to mammalian sequences in the KEGG database were discarded, and the subsequent data were used to quantify the abundance of each KEGG orthology group (KO) using the reads per kilobase of genome equivalents (RPKG) abundance statistic (125). Metagenomes were taxonomically annotated using MIDAS as described in reference 72.

### Statistical analyses and modeling.

The functional and taxonomic similarities between metagenomic samples were assessed using nonmetric multidimensional scaling (NMDS) as implemented through the nmds function in the labdsv R package (126). Ordinations were visualized using the ordiplot function in the vegan R package (127). For the functional similarity analysis, the vegdist function from the vegan R package quantified the Bray-Curtis dissimilarity based on KEGG module abundances. The taxonomic analysis used the generalized Unifrac (128) distances (alpha = 0.5), which were obtained by using the taxonomic tree from the Living Tree Project (129) and matching the genus and species components of tree leaf labels to the corresponding components of the MIDAS species labels in our data. Assessment of the significance of the clustering of samples in these ordination plots was conducted using PERMANOVA as implemented in the Adonis function in R.

The compound Poisson generalized linear mixed-effects model implemented in the cplm package in R (130) was used to find KEGG modules with significantly different time trends between groups while controlling for static differences between the lines and DNA extraction procedures (Qiagen versus MoBio). Random intercepts and slopes for both subjects and contributing KOs were used to capture variations between subjects and between genes while focusing on the large-scale shifts over the whole collection of abundance profiles contributing to a module. As described more thoroughly in Text S1 in the supplemental material, the general computational procedure consisted of forming subsets of the data with respect to each module’s relevant KO abundances and fitting a full model that described the RPKG abundance as a function of time, group, time by group interaction, sequencing kit, and random effects of each KO and individual. We then use two reduced models, eliminating first the interaction term and then the group term, to obtain *P* values via likelihood ratio tests. This is one of the recommended significance testing approaches for mixed models since it avoids using the approximations for the residual degrees of freedom that would be necessary to test significance via the t-statistic (66). To limit the number of modules tested, the input data were run through the MinPath Algorithm (131) to select a parsimonious set of modules based on the KOs present. The union of the individual parsimonious sets of all of the samples was used as the final set of tested modules. The approach of testing the dynamics of an entire module by fitting a single GLMM to a set of temporal abundances of multiple genes is modeled on the time course gene set analysis (TcGSA) method of Hejblum et al. (132), with the modification of using a different response distribution (the Tweedie compound Poisson distribution). Significant modules were selected at the 0.05 FDR threshold after controlling for multiple testing via the Benjamini-Hochberg (B-H) procedure. Species time trend differences were tested with the same approach, minus the grouping of multiple trajectories. Additional details of our modeling approach can be found in Text S1. All of the code used in this analysis is available at the following URL: https://github.com/slyalina/Mouse_IBD_2017_paper_supporting_code.

To differentiate functional changes occurring prior to immune activation, we fitted a second hinge regression to the abundances of modules that were found to have a significant time by group interaction in the main GLMM analysis. This second regression placed a break point at week 7, which represents the point at which immune activation initiated (Fig. 1). This allowed for two sets of slopes (before and after disease onset) and two sets of time by group interactions (representing deviations of DNR slopes from WT before-onset and after-onset slopes).

Alterations in the species trajectory curves were additionally tested with an alternate method aimed at highlighting differences in shape rather than slope. This method was an implementation of the FPCA-based difference in goodness-of-fit approach described previously in reference 133. The permutation-based *P* values from this analysis were B-H corrected, and species passing the 0.05 FDR threshold were retained.

To test the hypothesis that the distribution of the between-KO/within-KO dispersion decomposition statistics for all modules was significantly different from random grouping of functional trajectories (KOs into modules) but was not significantly different from random grouping of taxonomic trajectories (species into genera), we used the DISCO (71) nonparametric test, as well as the simulated null distributions that arise when generating random groupings of KOs and species, to obtain the real distributions of the test statistics in the two scenarios. We then performed a Kolmogorov-Smirnov test to compare the true distributions with their simulated counterparts.

### Accession number(s).

The metagenomic data that were generated and analyzed in this study are available in GenBank under BioProject number PRJNA397886.

## References

[1] Huttenhower C, Kostic AD, Xavier RJ 2014 Inflammatory bowel disease as a model for translating the microbiome. Immunity 40:843–854. doi:10.1016/j.immuni.2014.05.013. 24950204 PMC4135443

[2] Wlodarska M, Kostic AD, Xavier RJ 2015 An integrative view of microbiome-host interactions in inflammatory bowel diseases. Cell Host Microbe 17:577–591. doi:10.1016/j.chom.2015.04.008. 25974300 PMC4498258

[3] Ayres JS 2016 Cooperative microbial tolerance behaviors in host-microbiota mutualism. Cell 165:1323–1331. doi:10.1016/j.cell.2016.05.049. 27259146 PMC4903080

[4] Kostic AD, Xavier RJ, Gevers D 2014 The microbiome in inflammatory bowel disease: current status and the future ahead. Gastroenterology 146:1489–1499. doi:10.1053/j.gastro.2014.02.009. 24560869 PMC4034132

[5] Lees CW, Barrett JC, Parkes M, Satsangi J 2011 New IBD genetics: common pathways with other diseases. Gut 60:1739–1753. doi:10.1136/gut.2009.199679. 21300624

[6] Wirtz S, Neurath MF 2007 Mouse models of inflammatory bowel disease. Adv Drug Deliv Rev 59:1073–1083. doi:10.1016/j.addr.2007.07.003. 17825455

[7] Goodrich JK, Waters JL, Poole AC, Sutter JL, Koren O, Blekhman R, Beaumont M, Van Treuren W, Knight R, Bell JT, Spector TD, Clark AG, Ley RE 2014 Human genetics shape the gut microbiome. Cell 159:789–799. doi:10.1016/j.cell.2014.09.053. 25417156 PMC4255478

[8] Neuman MG, Nanau RM 2012 Inflammatory bowel disease: role of diet, microbiota, life style. Transl Res 160:29–44. doi:10.1016/j.trsl.2011.09.001. 22687961

[9] Bilski J, Mazur-Bialy A, Brzozowski B, Magierowski M, Zahradnik-Bilska J, Wójcik D, Magierowska K, Kwiecien S, Mach T, Brzozowski T 2016 Can exercise affect the course of inflammatory bowel disease? Experimental and clinical evidence. Pharmacol Rep 68:827–836. doi:10.1016/j.pharep.2016.04.009. 27255494

[10] Sartor RB, Mazmanian SK 2012 Intestinal microbes in inflammatory bowel diseases. Am J Gastroenterol Suppl 1:15–21. doi:10.1038/ajgsup.2012.4.

[11] Qin J, Li R, Raes J, Arumugam M, Burgdorf KS, Manichanh C, Nielsen T, Pons N, Levenez F, Yamada T, Mende DR, Li J, Xu J, Li S, Li D, Cao J, Wang B, Liang H, Zheng H, Xie Y, Tap J, Lepage P, Bertalan M, Batto JM, Hansen T, Le Paslier D, Linneberg A, Nielsen HB, Pelletier E, Renault P, Sicheritz-Ponten T, Turner K, Zhu H, Yu C, Li S, Jian M, Zhou Y, Li Y, Zhang X, Li S, Qin N, Yang H, Wang J, Brunak S, Dore J, Guarner F, Kristiansen K, Pedersen O, Parkhill J, et al. 2010 A human gut microbial gene catalogue established by metagenomic sequencing. Nature 464:59–65. doi:10.1038/nature08821. 20203603 PMC3779803

[12] Nielsen HB, Almeida M, Juncker AS, Rasmussen S, Li J, Sunagawa S, Plichta DR, Gautier L, Pedersen AG, Le Chatelier E, Pelletier E, Bonde I, Nielsen T, Manichanh C, Arumugam M, Batto JM, Quintanilha Dos Santos MB, Blom N, Borruel N, Burgdorf KS, Boumezbeur F, Casellas F, Doré J, Dworzynski P, Guarner F, Hansen T, Hildebrand F, Kaas RS, Kennedy S, Kristiansen K, Kultima JR, Léonard P, Levenez F, Lund O, Moumen B, Le Paslier D, Pons N, Pedersen O, Prifti E, Qin J, Raes J, Sorensen S, Tap J, Tims S, Ussery DW, Yamada T, MetaHIT Consortium, Renault P 2014 Identification and assembly of genomes and genetic elements in complex metagenomic samples without using reference genomes. Nat Biotechnol 32:822–828. doi:10.1038/nbt.2939. 24997787

[13] Morgan XC, Tickle TL, Sokol H, Gevers D, Devaney KL, Ward DV, Reyes JA, Shah SA, LeLeiko N, Snapper SB, Bousvaros A, Korzenik J, Sands BE, Xavier RJ, Huttenhower C 2012 Dysfunction of the intestinal microbiome in inflammatory bowel disease and treatment. Genome Biol 13:R79. doi:10.1186/gb-2012-13-9-r79. 23013615 PMC3506950

[14] Ott SJ, Musfeldt M, Wenderoth DF, Hampe J, Brant O, Fölsch UR, Timmis KN, Schreiber S 2004 Reduction in diversity of the colonic mucosa associated bacterial microflora in patients with active inflammatory bowel disease. Gut 53:685–693. doi:10.1136/gut.2003.025403. 15082587 PMC1774050

[15] Dubinsky M, Braun J 2015 Diagnostic and prognostic microbial biomarkers in inflammatory bowel diseases. Gastroenterology 149:1265–1274.e3. doi:10.1053/j.gastro.2015.08.006. 26284597 PMC5302020

[16] Nayfach S, Bradley PH, Wyman SK, Laurent TJ, Williams A, Eisen JA, Pollard KS, Sharpton TJ 2015 Automated and accurate estimation of gene family abundance from shotgun metagenomes. PLoS Comput Biol 11:e1004573. doi:10.1371/journal.pcbi.1004573. 26565399 PMC4643905

[17] Atarashi K, Tanoue T, Shima T, Imaoka A, Kuwahara T, Momose Y, Cheng G, Yamasaki S, Saito T, Ohba Y, Taniguchi T, Takeda K, Hori S, Ivanov II, Umesaki Y, Itoh K, Honda K 2011 Induction of colonic regulatory T cells by indigenous Clostridium species. Science 331:337–341. doi:10.1126/science.1198469. 21205640 PMC3969237

[18] Honda K, Littman DR 2012 The microbiome in infectious disease and inflammation. Annu Rev Immunol 30:759–795. doi:10.1146/annurev-immunol-020711-074937. 22224764 PMC4426968

[19] Chu H, Khosravi A, Kusumawardhani IP, Kwon AH, Vasconcelos AC, Cunha LD, Mayer AE, Shen Y, Wu WL, Kambal A, Targan SR, Xavier RJ, Ernst PB, Green DR, McGovern DP, Virgin HW, Mazmanian SK 2016 Gene-microbiota interactions contribute to the pathogenesis of inflammatory bowel disease. Science 352:1116–1120. doi:10.1126/science.aad9948. 27230380 PMC4996125

[20] David LA, Maurice CF, Carmody RN, Gootenberg DB, Button JE, Wolfe BE, Ling AV, Devlin AS, Varma Y, Fischbach MA, Biddinger SB, Dutton RJ, Turnbaugh PJ 2014 Diet rapidly and reproducibly alters the human gut microbiome. Nature 505:559–563. doi:10.1038/nature12820. 24336217 PMC3957428

[21] Carmody RN, Gerber GK, Luevano JM Jr, Gatti DM, Somes L, Svenson KL, Turnbaugh PJ 2015 Diet dominates host genotype in shaping the murine gut microbiota. Cell Host Microbe 17:72–84. doi:10.1016/j.chom.2014.11.010. 25532804 PMC4297240

[22] Rooks MG, Veiga P, Wardwell-Scott LH, Tickle T, Segata N, Michaud M, Gallini CA, Beal C, van Hylckama-Vlieg JE, Ballal SA, Morgan XC, Glickman JN, Gevers D, Huttenhower C, Garrett WS 2014 Gut microbiome composition and function in experimental colitis during active disease and treatment-induced remission. ISME J 8:1403–1417. doi:10.1038/ismej.2014.3. 24500617 PMC4069400

[23] Strober W, Fuss IJ, Blumberg RS 2002 The immunology of mucosal models of inflammation. Annu Rev Immunol 20:495–549. doi:10.1146/annurev.immunol.20.100301.064816. 11861611

[24] Bouma G, Strober W 2003 The immunological and genetic basis of inflammatory bowel disease. Nat Rev Immunol 3:521–533. doi:10.1038/nri1132. 12876555

[25] Hoffmann JC, Pawlowski NN, Kühl AA, Höhne W, Zeitz M 2002–2003 Animal models of inflammatory bowel disease: an overview. Pathobiology 70:121–130. 10.1159/000068143 12571415

[26] Mizoguchi A, Mizoguchi E, Bhan AK 2003 Immune networks in animal models of inflammatory bowel disease. Inflamm Bowel Dis 9:246–259. doi:10.1097/00054725-200307000-00005. 12902848

[27] Elson CO, Cong Y, McCracken VJ, Dimmitt RA, Lorenz RG, Weaver CT 2005 Experimental models of inflammatory bowel disease reveal innate, adaptive, and regulatory mechanisms of host dialogue with the microbiota. Immunol Rev 206:260–276. doi:10.1111/j.0105-2896.2005.00291.x. 16048554

[28] Valatas V, Vakas M, Kolios G 2013 The value of experimental models of colitis in predicting efficacy of biological therapies for inflammatory bowel diseases. Am J Physiol Gastrointest Liver Physiol 305:G763–G785. doi:10.1152/ajpgi.00004.2013. 23989010

[29] Valatas V, Bamias G, Kolios G 2015 Experimental colitis models: insights into the pathogenesis of inflammatory bowel disease and translational issues. Eur J Pharmacol 759:253–264. doi:10.1016/j.ejphar.2015.03.017. 25814256

[30] Gorelik L, Flavell RA 2000 Abrogation of TGFbeta signaling in T cells leads to spontaneous T cell differentiation and autoimmune disease. Immunity 12:171–181. doi:10.1016/S1074-7613(00)80170-3. 10714683

[31] Yamagiwa S, Gray JD, Hashimoto S, Horwitz DA 2001 A role for TGF-beta in the generation and expansion of CD4+CD25+ regulatory T cells from human peripheral blood. J Immunol 166:7282–7289. doi:10.4049/jimmunol.166.12.7282. 11390478

[32] Chen W, Jin W, Hardegen N, Lei KJ, Li L, Marinos N, McGrady G, Wahl SM 2003 Conversion of peripheral CD4+CD25- naive T cells to CD4+CD25+ regulatory T cells by TGF-beta induction of transcription factor Foxp3. J Exp Med 198:1875–1886. doi:10.1084/jem.20030152. 14676299 PMC2194145

[33] Schlenner SM, Weigmann B, Ruan Q, Chen Y, von Boehmer H 2012 Smad3 binding to the foxp3 enhancer is dispensable for the development of regulatory T cells with the exception of the gut. J Exp Med 209:1529–1535. doi:10.1084/jem.20112646. 22908322 PMC3428940

[34] Selvaraj RK, Geiger TL 2007 A kinetic and dynamic analysis of Foxp3 induced in T cells by TGF-beta. J Immunol 178:7667–7677. doi:10.4049/jimmunol.178.12.7667. 17548603

[35] Fantini MC, Becker C, Monteleone G, Pallone F, Galle PR, Neurath MF 2004 Cutting edge: TGF-beta induces a regulatory phenotype in CD4+CD25- T cells through Foxp3 induction and down-regulation of Smad7. J Immunol 172:5149–5153. doi:10.4049/jimmunol.172.9.5149. 15100250

[36] Huber S, Schramm C, Lehr HA, Mann A, Schmitt S, Becker C, Protschka M, Galle PR, Neurath MF, Blessing M 2004 Cutting edge: TGF-beta signaling is required for the in vivo expansion and immunosuppressive capacity of regulatory CD4+CD25+ T cells. J Immunol 173:6526–6531. doi:10.4049/jimmunol.173.11.6526. 15557141

[37] Marie JC, Letterio JJ, Gavin M, Rudensky AY 2005 TGF-beta1 maintains suppressor function and Foxp3 expression in CD4+CD25+ regulatory T cells. J Exp Med 201:1061–1067. doi:10.1084/jem.20042276. 15809351 PMC2213134

[38] Battaglia A, Buzzonetti A, Baranello C, Fanelli M, Fossati M, Catzola V, Scambia G, Fattorossi A 2013 Interleukin-21 (IL-21) synergizes with IL-2 to enhance T-cell receptor-induced human T-cell proliferation and counteracts IL-2/transforming growth factor-beta-induced regulatory T-cell development. Immunology 139:109–120. doi:10.1111/imm.12061. 23278180 PMC3634543

[39] Molinero LL, Miller ML, Evaristo C, Alegre ML 2011 High TCR stimuli prevent induced regulatory T cell differentiation in a NF-κB-dependent manner. J Immunol 186:4609–4617. doi:10.4049/jimmunol.1002361. 21411734 PMC3544303

[40] Bettelli E, Carrier Y, Gao W, Korn T, Strom TB, Oukka M, Weiner HL, Kuchroo VK 2006 Reciprocal developmental pathways for the generation of pathogenic effector TH17 and regulatory T cells. Nature 441:235–238. doi:10.1038/nature04753. 16648838

[41] Veldhoen M, Stockinger B 2006 TGFbeta1, a “jack of all trades”: the link with pro-inflammatory IL-17-producing T cells. Trends Immunol 27:358–361. doi:10.1016/j.it.2006.06.001. 16793343

[42] Li MO, Wan YY, Flavell RA 2007 T cell-produced transforming growth factor-beta1 controls T cell tolerance and regulates Th1- and Th17-cell differentiation. Immunity 26:579–591. doi:10.1016/j.immuni.2007.03.014. 17481928

[43] Gutcher I, Donkor MK, Ma Q, Rudensky AY, Flavell RA, Li MO 2011 Autocrine transforming growth factor-beta1 promotes *in vivo* Th17 cell differentiation. Immunity 34:396–408. doi:10.1016/j.immuni.2011.03.005. 21435587 PMC3690311

[44] Manel N, Unutmaz D, Littman DR 2008 The differentiation of human T(H)-17 cells requires transforming growth factor-beta and induction of the nuclear receptor RORgammat. Nat Immunol 9:641–649. doi:10.1038/ni.1610. 18454151 PMC2597394

[45] Yang L, Anderson DE, Baecher-Allan C, Hastings WD, Bettelli E, Oukka M, Kuchroo VK, Hafler DA 2008 IL-21 and TGF-beta are required for differentiation of human T(H)17 cells. Nature 454:350–352. doi:10.1038/nature07021. 18469800 PMC2760130

[46] Biancheri P, Giuffrida P, Docena GH, MacDonald TT, Corazza GR, Di Sabatino A 2014 The role of transforming growth factor (TGF)-beta in modulating the immune response and fibrogenesis in the gut. Cytokine Growth Factor Rev 25:45–55. doi:10.1016/j.cytogfr.2013.11.001. 24332927

[47] Di Giacinto C, Marinaro M, Sanchez M, Strober W, Boirivant M 2005 Probiotics ameliorate recurrent Th1-mediated murine colitis by inducing IL-10 and IL-10-dependent TGF-beta-bearing regulatory cells. J Immunol 174:3237–3246. doi:10.4049/jimmunol.174.6.3237. 15749854

[48] Feagins LA 2010 Role of transforming growth factor-beta in inflammatory bowel disease and colitis-associated colon cancer. Inflamm Bowel Dis 16:1963–1968. doi:10.1002/ibd.21281. 20848467

[49] Fuss IJ, Boirivant M, Lacy B, Strober W 2002 The interrelated roles of TGF-beta and IL-10 in the regulation of experimental colitis. J Immunol 168:900–908. doi:10.4049/jimmunol.168.2.900. 11777988

[50] Harrison OJ, Powrie FM 2013 Regulatory T cells and immune tolerance in the intestine. Cold Spring Harb Perspect Biol 5. doi:10.1101/cshperspect.a018341. PMC3685893 23818502

[51] Izcue A, Coombes JL, Powrie F 2009 Regulatory lymphocytes and intestinal inflammation. Annu Rev Immunol 27:313–338. doi:10.1146/annurev.immunol.021908.132657. 19302043

[52] Jarry A, Bossard C, Sarrabayrouse G, Mosnier JF, Laboisse CL 2011 Loss of interleukin-10 or transforming growth factor beta signaling in the human colon initiates a T-helper 1 response via distinct pathways. Gastroenterology 141:1887–1896.e1–2. doi:10.1053/j.gastro.2011.08.002. 21839042

[53] Kitani A, Fuss I, Nakamura K, Kumaki F, Usui T, Strober W 2003 Transforming growth factor (TGF)-beta1-producing regulatory T cells induce Smad-mediated interleukin 10 secretion that facilitates coordinated immunoregulatory activity and amelioration of TGF-beta1-mediated fibrosis. J Exp Med 198:1179–1188. doi:10.1084/jem.20030917. 14557415 PMC2194234

[54] Weiner HL 2001 Induction and mechanism of action of transforming growth factor-beta-secreting Th3 regulatory cells. Immunol Rev 182:207–214. doi:10.1034/j.1600-065X.2001.1820117.x. 11722636

[55] Franke A, McGovern DP, Barrett JC, Wang K, Radford-Smith GL, Ahmad T, Lees CW, Balschun T, Lee J, Roberts R, Anderson CA, Bis JC, Bumpstead S, Ellinghaus D, Festen EM, Georges M, Green T, Haritunians T, Jostins L, Latiano A, Mathew CG, Montgomery GW, Prescott NJ, Raychaudhuri S, Rotter JI, Schumm P, Sharma Y, Simms LA, Taylor KD, Whiteman D, Wijmenga C, Baldassano RN, Barclay M, Bayless TM, Brand S, Büning C, Cohen A, Colombel JF, Cottone M, Stronati L, Denson T, De Vos M, D’Inca R, Dubinsky M, Edwards C, Florin T, Franchimont D, Gearry R, Glas J, Van Gossum A, et al. 2010 Genome-wide meta-analysis increases to 71 the number of confirmed Crohn’s disease susceptibility loci. Nat Genet 42:1118–1125. doi:10.1038/ng.717. 21102463 PMC3299551

[56] Glocker EO, Kotlarz D, Boztug K, Gertz EM, Schäffer AA, Noyan F, Perro M, Diestelhorst J, Allroth A, Murugan D, Hätscher N, Pfeifer D, Sykora KW, Sauer M, Kreipe H, Lacher M, Nustede R, Woellner C, Baumann U, Salzer U, Koletzko S, Shah N, Segal AW, Sauerbrey A, Buderus S, Snapper SB, Grimbacher B, Klein C 2009 Inflammatory bowel disease and mutations affecting the interleukin-10 receptor. N Engl J Med 361:2033–2045. doi:10.1056/NEJMoa0907206. 19890111 PMC2787406

[57] McGovern DP, Gardet A, Torkvist L, Goyette P, Essers J, Taylor KD, Neale BM, Ong RT, Lagace C, Li C, Green T, Stevens CR, Beauchamp C, Fleshner PR, Carlson M, D'Amato M, Halfvarson J, Hibberd ML, Lordal M, Padyukov L, Andriulli A, Colombo E, Latiano A, Palmieri O, Bernard EJ, Deslandres C, Hommes DW, de Jong DJ, Stokkers PC, Weersma RK, Sharma Y, Silverberg MS, Cho JH, Wu J, Roeder K, Brant SR, Schumm LP, Duerr RH, Dubinsky MC, Glazer NL, Haritunians T, Ippoliti A, Melmed GY, Siscovick DS, Vasiliauskas EA, Targan SR, Annese V, Wijmenga C, Pettersson S, Rotter JI, et al. 2010 Genome-wide association identifies multiple ulcerative colitis susceptibility loci. Nat Genet 42:332–337. doi:10.1038/ng.549. 20228799 PMC3087600

[58] Naviglio S, Arrigo S, Martelossi S, Villanacci V, Tommasini A, Loganes C, Fabretto A, Vignola S, Lonardi S, Ventura A 2014 Severe inflammatory bowel disease associated with congenital alteration of transforming growth factor beta signaling. J Crohns Colitis 8:770–774. doi:10.1016/j.crohns.2014.01.013. 24486179

[59] Kang SS, Bloom SM, Norian LA, Geske MJ, Flavell RA, Stappenbeck TS, Allen PM 2008 An antibiotic-responsive mouse model of fulminant ulcerative colitis. PLoS Med 5:e41. doi:10.1371/journal.pmed.0050041. 18318596 PMC2270287

[60] Fowler SA, Ananthakrishnan AN, Gardet A, Stevens CR, Korzenik JR, Sands BE, Daly MJ, Xavier RJ, Yajnik V 2014 SMAD3 gene variant is a risk factor for recurrent surgery in patients with Crohn’s disease. J Crohns Colitis 8:845–851. doi:10.1016/j.crohns.2014.01.003. 24461721 PMC4237062

[61] Cho JH, Brant SR 2011 Recent insights into the genetics of inflammatory bowel disease. Gastroenterology 140:1704–1712. doi:10.1053/j.gastro.2011.02.046. 21530736 PMC4947143

[62] Henderson P, van Limbergen JE, Wilson DC, Satsangi J, Russell RK 2011 Genetics of childhood-onset inflammatory bowel disease. Inflamm Bowel Dis 17:346–361. doi:10.1002/ibd.21283. 20839313

[63] Ardizzone S, Bevivino G, Monteleone G 2016 Mongersen, an oral Smad7 antisense oligonucleotide, in patients with active Crohn’s disease. Therap Adv Gastroenterol 9:527–532. doi:10.1177/1756283X16636781. PMC4913329 27366221

[64] Laudisi F, Dinallo V, Di Fusco D, Monteleone G 2016 Smad7 and its potential as therapeutic target in inflammatory bowel diseases. Curr Drug Metab 17:303–306. doi:10.2174/1389200217666151210130103. 26651974

[65] Monteleone G, Boirivant M, Pallone F, MacDonald TT 2008 TGF-beta1 and Smad7 in the regulation of IBD. Mucosal Immunol 1(Suppl 1):S50–S53. doi:10.1038/mi.2008.55. 19079231

[66] Bolker BM, Brooks ME, Clark CJ, Geange SW, Poulsen JR, Stevens MH, White JS 2009 Generalized linear mixed models: a practical guide for ecology and evolution. Trends Ecol Evol 24:127–135. doi:10.1016/j.tree.2008.10.008. 19185386

[67] Székely GJ, Rizzo ML 2009 Brownian distance covariance. Ann Appl Stat 3:1236–1265. doi:10.1214/09-AOAS312. PMC2889501 20574547

[68] Bäumler AJ, Sperandio V 2016 Interactions between the microbiota and pathogenic bacteria in the gut. Nature 535:85–93. doi:10.1038/nature18849. 27383983 PMC5114849

[69] Byndloss MX, Rivera-Chávez F, Tsolis RM, Bäumler AJ 2017 How bacterial pathogens use type III and type IV secretion systems to facilitate their transmission. Curr Opin Microbiol 35:1–7. doi:10.1016/j.mib.2016.08.007. 27621139

[70] Mark Welch JL, Utter DR, Rossetti BJ, Mark Welch DB, Eren AM, Borisy GG 2014 Dynamics of tongue microbial communities with single-nucleotide resolution using oligotyping. Front Microbiol 5:568. doi:10.3389/fmicb.2014.00568. 25426106 PMC4224128

[71] Rizzo ML, Székely GJ 2010 DISCO analysis: a nonparametric extension of analysis of variance. Ann Appl Stat 4:1034–1055. doi:10.1214/09-AOAS245.

[72] Nayfach S, Rodriguez-Mueller B, Garud N, Pollard KS 2016 An integrated metagenomics pipeline for strain profiling reveals novel patterns of bacterial transmission and biogeography. Genome Res 26:1612–1625. doi:10.1101/gr.201863.115. 27803195 PMC5088602

[73] Prindiville TP, Sheikh RA, Cohen SH, Tang YJ, Cantrell MC, Silva J Jr 2000 Bacteroides fragilis enterotoxin gene sequences in patients with inflammatory bowel disease. Emerg Infect Dis 6:171–174. doi:10.3201/eid0602.000210. 10756151 PMC2640860

[74] Rabizadeh S, Rhee KJ, Wu S, Huso D, Gan CM, Golub JE, Wu X, Zhang M, Sears CL 2007 Enterotoxigenic Bacteroides fragilis: a potential instigator of colitis. Inflamm Bowel Dis 13:1475–1483. doi:10.1002/ibd.20265. 17886290 PMC3056612

[75] Bloom SM, Bijanki VN, Nava GM, Sun L, Malvin NP, Donermeyer DL, Dunne WM Jr, Allen PM, Stappenbeck TS 2011 Commensal Bacteroides species induce colitis in host-genotype-specific fashion in a mouse model of inflammatory bowel disease. Cell Host Microbe 9:390–403. doi:10.1016/j.chom.2011.04.009. 21575910 PMC3241010

[76] Conley MN, Wong CP, Duyck KM, Hord N, Ho E, Sharpton TJ 2016 Aging and serum MCP-1 are associated with gut microbiome composition in a murine model. PeerJ 4:e1854. doi:10.7717/peerj.1854. 27069796 PMC4824877

[77] Sartor RB 2008 Microbial influences in inflammatory bowel diseases. Gastroenterology 134:577–594. doi:10.1053/j.gastro.2007.11.059. 18242222

[78] Kaur N, Chen CC, Luther J, Kao JY 2011 Intestinal dysbiosis in inflammatory bowel disease. Gut Microbes 2:211–216. doi:10.4161/gmic.2.4.17863. 21983063

[79] Sellon RK, Tonkonogy S, Schultz M, Dieleman LA, Grenther W, Balish E, Rennick DM, Sartor RB 1998 Resident enteric bacteria are necessary for development of spontaneous colitis and immune system activation in interleukin-10-deficient mice. Infect Immun 66:5224–5231. 9784526 10.1128/iai.66.11.5224-5231.1998 PMC108652

[80] Garrett WS, Gallini CA, Yatsunenko T, Michaud M, DuBois A, Delaney ML, Punit S, Karlsson M, Bry L, Glickman JN, Gordon JI, Onderdonk AB, Glimcher LH 2010 Enterobacteriaceae act in concert with the gut microbiota to induce spontaneous and maternally transmitted colitis. Cell Host Microbe 8:292–300. doi:10.1016/j.chom.2010.08.004. 20833380 PMC2952357

[81] Chichlowski M, Westwood GS, Abraham SN, Hale LP 2010 Role of mast cells in inflammatory bowel disease and inflammation-associated colorectal neoplasia in IL-10-deficient mice. PLoS One 5:e12220. doi:10.1371/journal.pone.0012220. 20808919 PMC2923184

[82] Waidmann M, Bechtold O, Frick JS, Lehr HA, Schubert S, Dobrindt U, Loeffler J, Bohn E, Autenrieth IB 2003 Bacteroides vulgatus protects against Escherichia coli-induced colitis in gnotobiotic interleukin-2-deficient mice. Gastroenterology 125:162–177. doi:10.1016/S0016-5085(03)00672-3. 12851881

[83] Frank DN, St Amand AL, Feldman RA, Boedeker EC, Harpaz N, Pace NR 2007 Molecular-phylogenetic characterization of microbial community imbalances in human inflammatory bowel diseases. Proc Natl Acad Sci U S A 104:13780–13785. doi:10.1073/pnas.0706625104. 17699621 PMC1959459

[84] Sokol H, Seksik P, Rigottier-Gois L, Lay C, Lepage P, Podglajen I, Marteau P, Doré J 2006 Specificities of the fecal microbiota in inflammatory bowel disease. Inflamm Bowel Dis 12:106–111. doi:10.1097/01.MIB.0000200323.38139.c6. 16432374

[85] Peterson DA, Frank DN, Pace NR, Gordon JI 2008 Metagenomic approaches for defining the pathogenesis of inflammatory bowel diseases. Cell Host Microbe 3:417–427. doi:10.1016/j.chom.2008.05.001. 18541218 PMC2872787

[86] Sokol H, Seksik P, Furet JP, Firmesse O, Nion-Larmurier I, Beaugerie L, Cosnes J, Corthier G, Marteau P, Doré J 2009 Low counts of Faecalibacterium prausnitzii in colitis microbiota. Inflamm Bowel Dis 15:1183–1189. doi:10.1002/ibd.20903. 19235886

[87] Pasolli E, Truong DT, Malik F, Waldron L, Segata N 2016 Machine learning meta-analysis of large metagenomic datasets: tools and biological insights. PLoS Comput Biol 12:e1004977. doi:10.1371/journal.pcbi.1004977. 27400279 PMC4939962

[88] Casellas F, Borruel N, Papo M, Guarner F, Antolín M, Videla S, Malagelada JR 1998 Antiinflammatory effects of enterically coated amoxicillin-clavulanic acid in active ulcerative colitis. Inflamm Bowel Dis 4:1–5. doi:10.1097/00054725-199802000-00001. 9552221

[89] Rietdijk ST, D’Haens GR 2013 Recent developments in the treatment of inflammatory bowel disease. J Dig Dis 14:282–287. doi:10.1111/1751-2980.12048. 23419117

[90] Gusils C, Morata V, González S 2004 Determination of bacterial adhesion to intestinal mucus. Methods Mol Biol 268:411–415. doi:10.1385/1-59259-766-1:411. 15156051

[91] Murch SH, MacDonald TT, Walker-Smith JA, Levin M, Lionetti P, Klein NJ 1993 Disruption of sulphated glycosaminoglycans in intestinal inflammation. Lancet 341:711–714. doi:10.1016/0140-6736(93)90485-Y. 8095623

[92] Benjdia A, Martens EC, Gordon JI, Berteau O 2011 Sulfatases and a radical S-adenosyl-l-methionine (AdoMet) enzyme are key for mucosal foraging and fitness of the prominent human gut symbiont, Bacteroides thetaiotaomicron. J Biol Chem 286:25973–25982. doi:10.1074/jbc.M111.228841. 21507958 PMC3138274

[93] Ulmer JE, Vilén EM, Namburi RB, Benjdia A, Beneteau J, Malleron A, Bonnaffé D, Driguez PA, Descroix K, Lassalle G, Le Narvor C, Sandström C, Spillmann D, Berteau O 2014 Characterization of glycosaminoglycan (GAG) sulfatases from the human gut symbiont Bacteroides thetaiotaomicron reveals the first GAG-specific bacterial endosulfatase. J Biol Chem 289:24289–24303. doi:10.1074/jbc.M114.573303. 25002587 PMC4148858

[94] Heuston S, Begley M, Gahan CG, Hill C 2012 Isoprenoid biosynthesis in bacterial pathogens. Microbiology 158:1389–1401. doi:10.1099/mic.0.051599-0. 22466083

[95] Pereira C, Grácio D, Teixeira JP, Magro F 2015 Oxidative stress and DNA damage: implications in inflammatory bowel disease. Inflamm Bowel Dis 21:2403–2417. doi:10.1097/MIB.0000000000000506. 26193347

[96] Steiner TS 2007 How flagellin and Toll-like receptor 5 contribute to enteric infection. Infect Immun 75:545–552. doi:10.1128/IAI.01506-06. 17118981 PMC1828527

[97] Cullender TC, Chassaing B, Janzon A, Kumar K, Muller CE, Werner JJ, Angenent LT, Bell ME, Hay AG, Peterson DA, Walter J, Vijay-Kumar M, Gewirtz AT, Ley RE 2013 Innate and adaptive immunity interact to quench microbiome flagellar motility in the gut. Cell Host Microbe 14:571–581. doi:10.1016/j.chom.2013.10.009. 24237702 PMC3920589

[98] Michielan A, D’Incà R 2015 Intestinal permeability in inflammatory bowel disease: pathogenesis, clinical evaluation, and therapy of leaky gut. Mediators Inflamm 2015:628157. doi:10.1155/2015/628157. 26582965 PMC4637104

[99] Nugent SG, Kumar D, Rampton DS, Evans DF 2001 Intestinal luminal pH in inflammatory bowel disease: possible determinants and implications for therapy with aminosalicylates and other drugs. Gut 48:571–577. doi:10.1136/gut.48.4.571. 11247905 PMC1728243

[100] Wang Z, Klipfell E, Bennett BJ, Koeth R, Levison BS, Dugar B, Feldstein AE, Britt EB, Fu X, Chung YM, Wu Y, Schauer P, Smith JD, Allayee H, Tang WH, DiDonato JA, Lusis AJ, Hazen SL 2011 Gut flora metabolism of phosphatidylcholine promotes cardiovascular disease. Nature 472:57–63. doi:10.1038/nature09922. 21475195 PMC3086762

[101] Schicho R, Marsche G, Storr M 2015 Cardiovascular complications in inflammatory bowel disease. Curr Drug Targets 16:181–188. doi:10.2174/1389450116666150202161500. 25642719 PMC4366573

[102] Andersen NN, Jess T 2014 Risk of cardiovascular disease in inflammatory bowel disease. World J Gastrointest Pathophysiol 5:359–365. doi:10.4291/wjgp.v5.i3.359. 25133036 PMC4133533

[103] Gootenberg DB, Paer JM, Luevano JM, Kwon DS 2017 HIV-associated changes in the enteric microbial community: potential role in loss of homeostasis and development of systemic inflammation. Curr Opin Infect Dis 30:31–43. doi:10.1097/QCO.0000000000000341. 27922852 PMC5325247

[104] Trøseid M, Manner IW, Pedersen KK, Haissman JM, Kvale D, Nielsen SD 2014 Microbial translocation and cardiometabolic risk factors in HIV infection. AIDS Res Hum Retroviruses 30:514–522. doi:10.1089/aid.2013.0280. 24521167 PMC4046217

[105] Nobles CL, Maresso AW 2011 The theft of host heme by Gram-positive pathogenic bacteria. Metallomics 3:788–796. doi:10.1039/c1mt00047k. 21725569

[106] Anzaldi LL, Skaar EP 2010 Overcoming the heme paradox: heme toxicity and tolerance in bacterial pathogens. Infect Immun 78:4977–4989. doi:10.1128/IAI.00613-10. 20679437 PMC2981329

[107] Gasche C, Lomer MC, Cavill I, Weiss G 2004 Iron, anaemia, and inflammatory bowel diseases. Gut 53:1190–1197. doi:10.1136/gut.2003.035758. 15247190 PMC1774131

[108] Kaitha S, Bashir M, Ali T 2015 Iron deficiency anemia in inflammatory bowel disease. World J Gastrointest Pathophysiol 6:62–72. doi:10.4291/wjgp.v6.i3.62. 26301120 PMC4540708

[109] Williams HR, Cox IJ, Walker DG, North BV, Patel VM, Marshall SE, Jewell DP, Ghosh S, Thomas HJ, Teare JP, Jakobovits S, Zeki S, Welsh KI, Taylor-Robinson SD, Orchard TR 2009 Characterization of inflammatory bowel disease with urinary metabolic profiling. Am J Gastroenterol 104:1435–1444. doi:10.1038/ajg.2009.175. 19491857

[110] Williams HR, Cox IJ, Walker DG, Cobbold JF, Taylor-Robinson SD, Marshall SE, Orchard TR 2010 Differences in gut microbial metabolism are responsible for reduced hippurate synthesis in Crohn’s disease. BMC Gastroenterol 10:108. doi:10.1186/1471-230X-10-108. 20849615 PMC2954941

[111] Radogna F, Diederich M, Ghibelli L 2010 Melatonin: a pleiotropic molecule regulating inflammation. Biochem Pharmacol 80:1844–1852. doi:10.1016/j.bcp.2010.07.041. 20696138

[112] Paulose JK, Wright JM, Patel AG, Cassone VM 2016 Human gut bacteria are sensitive to melatonin and express endogenous circadian rhythmicity. PLoS One 11:e0146643. doi:10.1371/journal.pone.0146643. 26751389 PMC4709092

[113] Paiva CN, Bozza MT 2014 Are reactive oxygen species always detrimental to pathogens? Antioxid Redox Signal 20:1000–1037. doi:10.1089/ars.2013.5447. 23992156 PMC3924804

[114] Preston A, Mandrell RE, Gibson BW, Apicella MA 1996 The lipooligosaccharides of pathogenic gram-negative bacteria. Crit Rev Microbiol 22:139–180. doi:10.3109/10408419609106458. 8894399

[115] Cárdenas L, Domínguez J, Santana O, Quinto C 1996 The role of the nodI and nodJ genes in the transport of Nod metabolites in Rhizobium etli. Gene 173:183–187. doi:10.1016/0378-1119(96)00166-7. 8964496

[116] Vázquez M, Santana O, Quinto C 1993 The NodL and NodJ proteins from Rhizobium and Bradyrhizobium strains are similar to capsular polysaccharide secretion proteins from gram-negative bacteria. Mol Microbiol 8:369–377. doi:10.1111/j.1365-2958.1993.tb01580.x. 8316086

[117] Funderburg NT, Stubblefield Park SR, Sung HC, Hardy G, Clagett B, Ignatz-Hoover J, Harding CV, Fu P, Katz JA, Lederman MM, Levine AD 2013 Circulating CD4(+) and CD8(+) T cells are activated in inflammatory bowel disease and are associated with plasma markers of inflammation. Immunology 140:87–97. doi:10.1111/imm.12114. 23600521 PMC3809709

[118] Integrative HMP (iHMP) Research Network Consortium 2014 The Integrative Human Microbiome Project: dynamic analysis of microbiome-host omics profiles during periods of human health and disease. Cell Host Microbe 16:276–289. doi:10.1016/j.chom.2014.08.014. 25211071 PMC5109542

[119] Human Microbiome Project Consortium 2012 Structure, function and diversity of the healthy human microbiome. Nature 486:207–214. doi:10.1038/nature11234. 22699609 PMC3564958

[120] Sharpton TJ 2017 A high-throughput and modular workflow to quality control shotgun metagenomic DNA The sequence libraries. https://zenodo.org/record/834783.

[121] Schmieder R, Edwards R 2011 Quality control and preprocessing of metagenomic datasets. Bioinformatics 27:863–864. doi:10.1093/bioinformatics/btr026. 21278185 PMC3051327

[122] Rotmistrovsky K, Agarwala R 2011 BMTagger: Best Match Tagger for removing human reads from metagenomics data sets. ftp://ftp.ncbi.nlm.nih.gov/pub/agarwala/bmtagger/.

[123] Hyatt D, Chen GL, Locascio PF, Land ML, Larimer FW, Hauser LJ 2010 Prodigal: prokaryotic gene recognition and translation initiation site identification. BMC Bioinformatics 11:119. doi:10.1186/1471-2105-11-119. 20211023 PMC2848648

[124] Zhao Y, Tang H, Ye Y 2012 RAPSearch2: a fast and memory-efficient protein similarity search tool for next-generation sequencing data. Bioinformatics 28:125–126. doi:10.1093/bioinformatics/btr595. 22039206 PMC3244761

[125] Nayfach S, Pollard KS 2015 Average genome size estimation improves comparative metagenomics and sheds light on the functional ecology of the human microbiome. Genome Biol 16:51. doi:10.1186/s13059-015-0611-7. 25853934 PMC4389708

[126] Roberts DW 2016 labdsv: ordination and multivariate analysis for ecology. https://cran.r-project.org/web/packages/labdsv/.

[127] Jari Oksanen FGB, Friendly M, Kindt R, Legendre P, McGlinn D, Minchin PR, O’Hara RB, Simpson GL, Peter Solymos M, Stevens HH, Szoecs E, Wagner H 2017 vegan: community ecology package. https://cran.r-project.org/web/packages/vegan/.

[128] Chen J, Bittinger K, Charlson ES, Hoffmann C, Lewis J, Wu GD, Collman RG, Bushman FD, Li H 2012 Associating microbiome composition with environmental covariates using generalized UniFrac distances. Bioinformatics 28:2106–2113. doi:10.1093/bioinformatics/bts342. 22711789 PMC3413390

[129] Yarza P, Ludwig W, Euzéby J, Amann R, Schleifer KH, Glöckner FO, Rosselló-Móra R 2010 Update of the All-Species Living Tree Project based on 16S and 23S rRNA sequence analyses. Syst Appl Microbiol 33:291–299. doi:10.1016/j.syapm.2010.08.001. 20817437

[130] Zhang Y 2013 Likelihood-based and Bayesian methods for Tweedie compound Poisson linear mixed models. Stat Comput 23:743–757. doi:10.1007/s11222-012-9343-7.

[131] Ye Y, Doak TG 2009 A parsimony approach to biological pathway reconstruction/inference for genomes and metagenomes. PLoS Comput Biol 5:e1000465. doi:10.1371/journal.pcbi.1000465. 19680427 PMC2714467

[132] Hejblum BP, Skinner J, Thiébaut R 2015 Time course gene set analysis for longitudinal gene expression data. PLoS Comput Biol 11:e1004310. doi:10.1371/journal.pcbi.1004310. 26111374 PMC4482329

[133] Wu S, Wu H 2013 More powerful significant testing for time course gene expression data using functional principal component analysis approaches. BMC Bioinformatics 14:6. doi:10.1186/1471-2105-14-6. 23323795 PMC3617096

[134] Lenth RV 2016 Least-squares means: the R package lsmeans. J Stat Softw 69:33 https://www.jstatsoft.org/v069/i01.

