# Supplementary material for: Development of Inflammatory Bowel Disease Is Linked to a Longitudinal Restructuring of the Gut Metagenome in Mice
Source: mSystems. 2017 Sep 5;2(5):e00036-17. doi: 10.1128/mSystems.00036-17 (PMC5585689; doi:10.1128/mSystems.00036-17)
Supplement: TEXT S1 [file sys005172134s5.docx]

For each KEGG module, the *cpglmm* function in the *cplm* R package was used to fit three models with the following regression formulas specified in Wilkinson notation:

$$Abundance \sim time+kit+\left( 1+time | subject \right)+\left( 1+time | KO \right)$$

$$Abundance \sim time+group+ kit+\left( 1+time | subject \right)+\left( 1+time | KO \right)$$

$$Abundance \sim time+group+group:time+kit+\left( 1+time | subject \right)+\left( 1+time | KO \right)$$

The introduction of random intercepts and slopes for the contributing KOs allows us to look at whole module shifts while accounting for deviations caused by inter-gene variation. As these models are nested and not fit via REML, the statistical significance of adding a group effect and a group:time effect can be obtained from a likelihood ratio test performed by the *anova* function also in the *cplm* package.

To perform the segmented GLMM analysis, two helper functions were defined to model the “hinge” at week 7. These were:

b1 = $\left\{ \begin{aligned} 7-x; if x \leq7 \\ 0; if x>7 \end{aligned} \right.$ and b2=$\left\{ \begin{aligned} 0; if x \leq7 \\ x-7; if x>7 \end{aligned} \right.$

Using these auxiliary functions the full regression formula was respecified:

$$Abundance \sim\left( b1\left( time \right)+b2\left( time \right) \right)*group+kit+\left( 1+b1\left( time \right)+b2\left( time \right) | subject \right)+\left( 1+b1\left( time \right)+b2\left( time \right) | KO \right)$$

Significance of the newly found segmented slope coefficients was assessed via their t-values.
